# Reversible Interlobular Septal ‘Pearls’ Associated with Takotsubo Cardiomyopathy and Heart Failure

**DOI:** 10.5334/jbsr.4081

**Published:** 2025-09-24

**Authors:** Maxence Van Belle, Stéphane B Van den Broeck

**Affiliations:** 1 Department of Diagnostic Radiology, Clinique St Luc Bouge (Namur), Belgium

**Keywords:** takotsubo cardiomyopathy, acute heart failure, interstitial lung nodules, thoracic CT pattern

## Abstract

*Teaching point:* Transient interlobular septal nodules displaying a characteristic branched ‘pearl-like’ pattern may reflect acute pulmonary lymphatic overload and should not be mistaken for other common pathological processes.

## Case History

An 85‑year‑old female presented to the emergency department with acute‑onset epigastric and left‑sided chest pain associated with grade 4 dyspnea following mild physical exertion. Her medical history is notable for a left bundle branch block, arterial hypertension, and hypercholesterolemia. The initial workup revealed elevated troponin levels (70 ng/L, which increased to 218 ng/L in a few hours), no ST‑segment elevation, and a normal chest X‑ray. The patient was subsequently referred for a contrast‑enhanced thoracoabdominal computed tomography (CT) to exclude any acute thoracoabdominal surgical condition.

**Figure 1:**
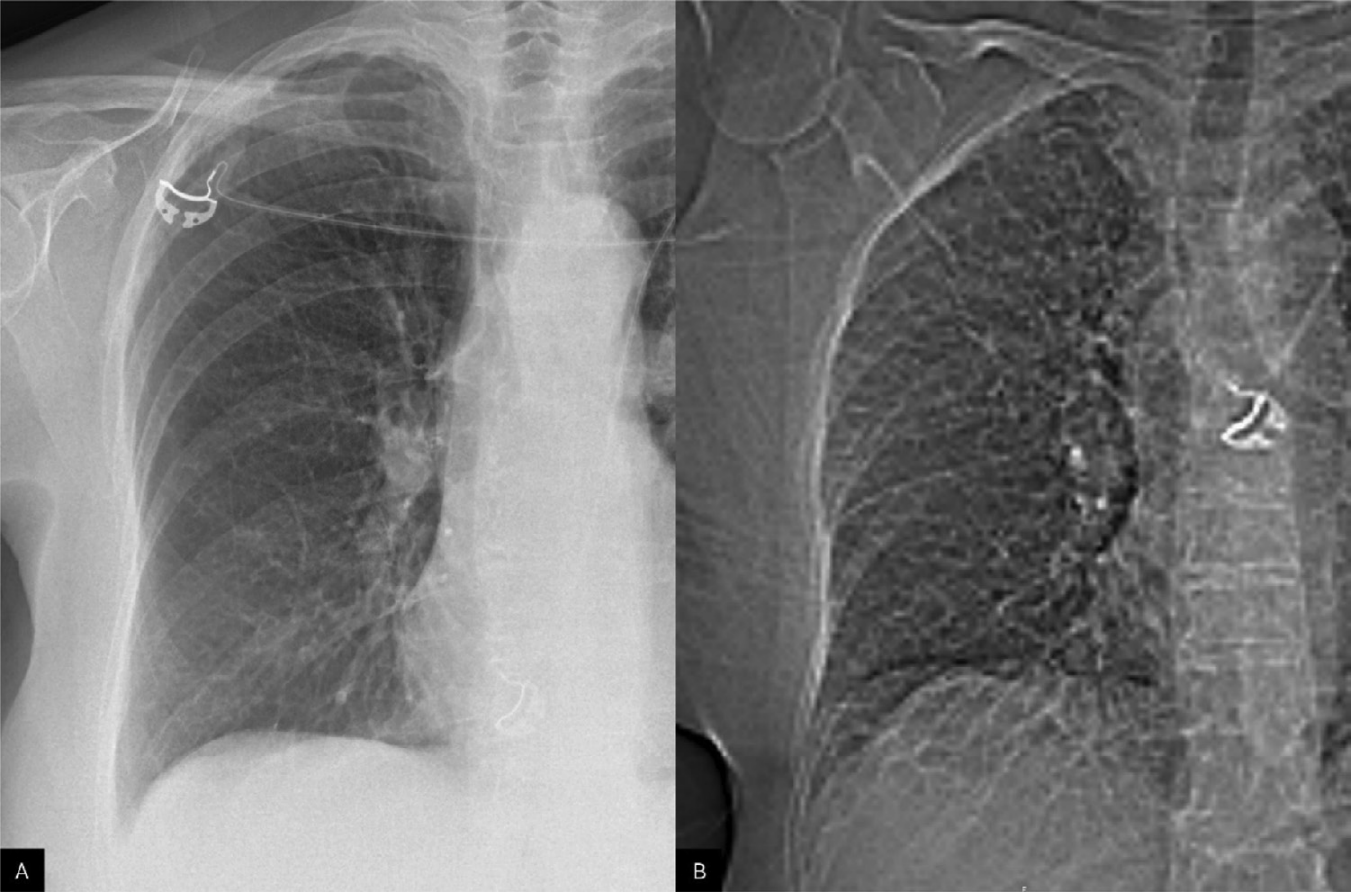
Comparison between the admission chest X‑ray **(A)** and the scout view from the CT scan performed two hours later **(B)**, showing the onset of linear parenchymal opacities in the peripheral region of the right lung field.

**Figure 2:**
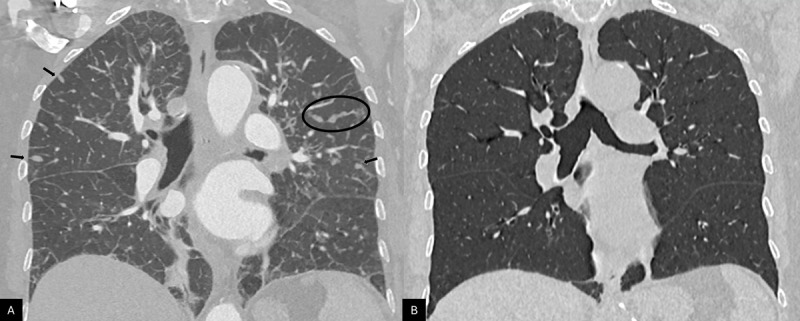
Comparison between a coronal CT view obtained on the day of admission **(A)** and a coronal CT view performed eight weeks later **(B)**, showing multiple diffuse ovoid and branched nodules (black arrows) distributed along thickened interlobular septa (black circle), which resolved on follow‑up imaging.

**Figure 3:**
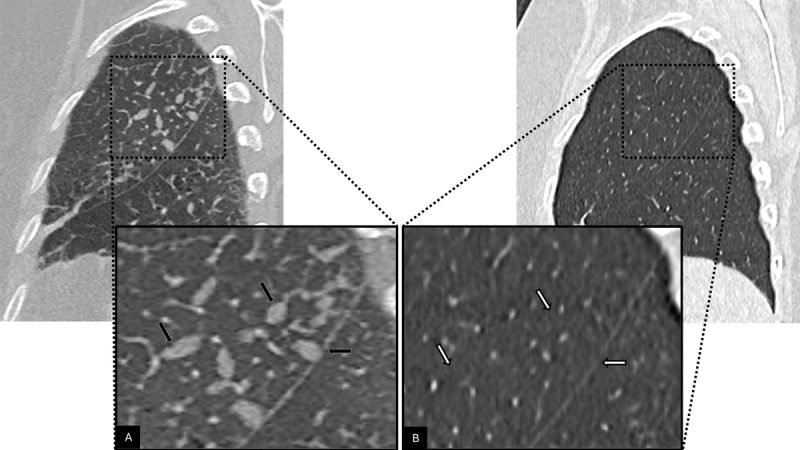
Sagittal CT views at admission **(A)** and at eight‑week follow‑up **(B)**, with magnified views of selected regions of interest, showing the previously observed ovoid and branched nodules (black arrows) and their resolution over time (white arrows).

CT revealed signs of acute cardiac dysfunction with severe interstitial congestion. Compared to the chest X‑ray performed two hours earlier, the CT scout view showed a sudden onset of peripheral interstitial lung attenuation (Figure 1A and 1B). Additional CT findings included peribronchovascular cuffing and interlobular septal thickening (interstitial edema), some diffuse ground‑glass opacities (alveolar edema), a diffuse infiltration of the mediastinal fat with some enlarged hypodense mediastinal lymph nodes, and a mild bilateral pleural effusion. In addition, multiple diffuse ovoid and branched nodules of water density were observed along the thickened interlobular septa (Figures 2A and 3A). These nodules were not suggestive of the most common etiologies responsible for diffuse pulmonary nodules, including metastatic disease or infectious etiologies.

Emergency coronary angiography, with ventriculography and cardiac ultrasound, revealed no significant coronary artery stenosis but identified apical akinesia of the left ventricle, consistent with Takotsubo syndrome. Medical treatment consisting of a single antiplatelet therapy was initiated, with a favorable clinical course allowing discharge after three days. An unenhanced chest CT performed eight weeks later demonstrated complete resolution of the previously identified abnormalities, in particular the elongated nodules (Figures 2B and 3B).

## Comments

These nodules are thought to represent transient interstitial lymphatic ectasia most likely resulting from an acute volume overload of the pulmonary circulation. This rare imaging pattern has previously been described as ‘lymphatic pearls’ in a single published case of acute left heart failure due to sick sinus syndrome and mitral valve prolapse [1]. In both cases, branched nodules appeared exclusively along the thickened interlobular septa, matched fluid density, were not present on prior imaging studies, and resolved completely following treatment of heart failure.

It is not uncommon for patients presenting with acute cardiac dysfunction to undergo chest CT to exclude an alternative life‑threatening diagnosis. The attention of radiologists is attracted to this benign and reversible imaging pattern to avoid misinterpretation. Moreover, recognizing this pattern associated with other signs of cardiogenic pulmonary edema may contribute to the rapid identification of acute cardiac dysfunction on CT.
